# Provider and household costs of *Plasmodium vivax* malaria episodes: a multicountry comparative analysis of primary trial data

**DOI:** 10.2471/BLT.18.226688

**Published:** 2019-09-27

**Authors:** Angela Devine, Ayodhia P Pasaribu, Tedlla Teferi, Huong-Thu Pham, Ghulam Rahim Awab, Febrina Contantia, Thuy-Nhien Nguyen, Viet-Thanh Ngo, Tinh-Hien Tran, Asrat Hailu, Kim Gilchrist, Justin A Green, Gavin CKW Koh, Kamala Thriemer, Walter RJ Taylor, Nicholas PJ Day, Ric N Price, Yoel Lubell

**Affiliations:** a Menzies School of Health Research, Charles Darwin University, PO Box 41096, Casuarina, Northern Territory 0811, Australia.; b Medical Faculty, Universitas Sumatera Utara, Medan, Indonesia.; c Arba Minch Hospital, Arba Minch, Ethiopia.; d Oxford University Clinical Research Unit, Hospital for Tropical Diseases, Ho Chi Minh City, Viet Nam.; e Medical Faculty, Nangarhar University, Kabul, Afghanistan.; f Tanjung Leidong Health Center, Tanjung Leidong, Indonesia.; g Oxford University Clinical Research Unit, University of Oxford, Oxford, England.; h School of Medicine, Addis Ababa University, Addis Ababa, Ethiopia.; i GlaxoSmithKline, Collegeville, Pennsylvania, United States of America.; j GlaxoSmithKline Research & Development, Uxbridge, England.; k Mahidol-Oxford Tropical Medicine Research Unit, Mahidol University, Bangkok, Thailand.

## Abstract

**Objective:**

To determine household and health-care provider costs associated with *Plasmodium vivax* infection across a range of endemic settings.

**Methods:**

We collected cost data alongside three multicentre clinical trials of *P. vivax* treatment in Afghanistan, Brazil, Colombia, Ethiopia, Indonesia, Philippines, Peru, Thailand and Viet Nam conducted between April 2014 to December 2017. We derived household costs from trial participant surveys administered at enrolment and again 2 weeks later to determine the costs of treatment and transportation, and the number of days that patients and their household caregivers were unable to undertake their usual activities. We determined costs of routine care by health-care providers by micro-costing the resources used to diagnose and treat *P. vivax* at the study sites.

**Findings:**

The mean total household costs ranged from 8.7 United States dollars (US$; standard deviation, SD: 4.3) in Afghanistan to US$ 254.7 (SD: 148.4) in Colombia. Across all countries, productivity losses were the largest household cost component, resulting in mean indirect costs ranging from US$ 5.3 (SD: 3.0) to US$ 220.8 (SD: 158.40). The range of health-care provider costs for routine care was US$ 3.6–6.6. The cost of administering a glucose-6-phosphate-dehydrogenase rapid diagnostic test, ranged from US$ 0.9 to 13.5, consistently lower than the costs of the widely-used fluorescent spot test (US$ 6.3 to 17.4).

**Conclusion:**

An episode of *P. vivax* malaria results in high costs to households. The costs of diagnosing and treating *P. vivax* are important inputs for future cost–effectiveness analyses to ensure optimal allocation of resources for malaria elimination.

## Résumé

**Objectif:**

Déterminer les coûts, pour les ménages et les prestataires de soins, associés à l'infection par *Plasmodium vivax* dans différentes zones endémiques.

**Méthodes:**

Nous avons recueilli des données sur les coûts à l'occasion de trois essais cliniques multicentres relatifs au traitement contre *P. vivax* menés en Afghanistan, au Brésil, en Colombie, en Éthiopie, en Indonésie, aux Philippines, au Pérou, en Thaïlande et au Viet Nam entre avril 2014 et décembre 2017. Nous avons calculé les coûts pour les ménages à partir d'enquêtes réalisées auprès des participants aux essais, lors de leur admission puis 2 semaines plus tard, afin de déterminer les coûts de traitement et de transport ainsi que le nombre de jours où les patients et leurs aidants familiaux n'avaient pu accomplir leurs activités habituelles. Nous avons déterminé le coût des soins courants par les prestataires de soins en calculant le coût individuel des ressources utilisées pour diagnostiquer et traiter *P. vivax* sur le site des études.

**Résultats:**

Les coûts totaux moyens pour les ménages allaient de 8,7 dollars des États-Unis ($ US; écart type, ET: 4,3) en Afghanistan à 254,7 $ US (ET: 148,4) en Colombie. Tous pays confondus, les pertes de productivité étaient la principale composante des coûts pour les ménages, puisqu'elles entraînaient des coûts indirects moyens allant de 5,3 $ US (ET: 3,0) à 220,8 $ US (ET: 158,40). En ce qui concerne les prestataires de soins, le coût des soins courants allait de 3,6 à 6,6 $ US. Le coût de réalisation d'un test de diagnostic rapide basé sur le glucose-6-phosphate-déshydrogénase variait de 0,9 $ US à 13,5 $ US, un coût toujours inférieur à celui du très répandu test par fluorescence (6,3 $ US à 17,4 $ US).

**Conclusion:**

Un épisode de paludisme à *P. vivax* entraîne des coûts élevés pour les ménages. Connaître les coûts du diagnostic et du traitement de *P. vivax* sera fort utile aux futures analyses coût-efficacité. Cela permettra d'affecter les ressources de manière optimale en vue d'éradiquer le paludisme.****

## Resumen

**Objetivo:**

Determinar los costos del hogar y de los proveedores de atención de la salud asociados con la infección por *Plasmodium vivax* en una variedad de ámbitos endémicos.

**Métodos:**

Se recopilaron datos de costos junto con tres ensayos clínicos multicéntricos del tratamiento con *P. vivax* en Afganistán, Brasil, Colombia, Etiopía, Filipinas, Indonesia, Perú, Tailandia y Vietnam realizados entre abril de 2014 y diciembre de 2017. Los costos del hogar se derivaron de las encuestas de los participantes del ensayo administradas en el momento de la inscripción y de nuevo dos semanas después para determinar los costos del tratamiento y del transporte, así como el número de días en que los pacientes y los cuidadores en el hogar no pudieron llevar a cabo sus actividades habituales. Se determinaron los costos de la atención de rutina por parte de los proveedores de atención de la salud mediante el microcosto de los recursos utilizados para diagnosticar y tratar el *P. vivax* en los centros de estudio.

**Resultados:**

Los costos totales promedio de los hogares oscilaron entre 8,7 dólares estadounidenses (USD; desviación estándar, DE: 4,3) en Afganistán y 254,7 USD (DE: 148,4) en Colombia. En todos los países, las pérdidas de productividad fueron el mayor componente del costo del hogar, lo que dio lugar a costos indirectos promedios que oscilaron entre 5,3 y 220,8 USD. El rango de los costos de los proveedores de atención de la salud para la atención de rutina fue de 3,6 a 6,6 USD. El costo por administrar una prueba de diagnóstico rápido de la glucosa-6-fosfato-deshidrogenasa, varió de 0,9 a 13,5 USD, consistentemente más bajo que los costos de la prueba de fluorescencia aleatoria ampliamente utilizada (6,3 a 17,4 USD).

**Conclusión:**

Un episodio de malaria por *P. vivax* tiene como resultado un alto costo para el hogar. Los costos del diagnóstico y tratamiento de *P. vivax* son insumos importantes para futuros análisis de costo-efectividad que aseguren una asignación óptima de recursos para la eliminación de la malaria.

## ملخص

**الغرض:**

تحديد تكاليف مزود الرعاية الصحية والتكاليف الأسرية المرتبطة بعدوى بلازموديوم فيفاكس عبر مجموعة من الأوضاع الوبائية.

**الطريقة:**

قمنا بتجميع بيانات التكلفة إلى جانب ثلاث تجارب سريرية متعددة المراكز لعلاج بلازموديوم فيفاكس في كل من إثيوبيا وأفغانستان وإندونيسيا والبرازيل والفلبين وبيرو وتايلند وفييت نام وكولومبيا، تمت ما بين أبريل/نيسان 2014 إلى ديسمبر/كانون أول 2017. وحصلنا على التكاليف الأسرية من مسوح للمشاركين في التجربة تم إجراؤها عند التسجيل، ثم مرة أخرى بعد أسبوعين من تحديد تكاليف العلاج والنقل، وعدد الأيام التي لم يتمكن فيها المرضى ومقدمو الرعاية المنزلية من القيام بأنشطتهم المعتادة. قمنا بتحديد تكاليف الرعاية الروتينية من جانب مقدمي الرعاية الصحية عن طريق تقدير التكاليف الدقيقة للموارد المستخدمة لتشخيص وعلاج بلازموديوم فيفاكس في مواقع الدراسة.

النتائج **** تراوح متوسط ​​إجمالي تكاليف الأسرة من 8.7 دولاراً أمريكياً (دولار أمريكي؛ الانحراف المعياري: 4.3) في أفغانستان إلى 254.7 دولاراً أمريكياً (الانحراف المعياري: 148.4) في كولومبيا. في كل البلدان، كانت خسائر الإنتاجية هي أكبر عنصر في التكلفة الأسرية، مما أسفر عن أن يتراوح متوسط التكاليف غير المباشرة من 5.3 دولار أمريكي (الانحراف المعياري: 3.0) إلى 220.8 دولاراً أمريكياً (الانحراف المعياري: 158.40). كان نطاق تكاليف مقدم الرعاية الصحية للرعاية الروتينية يتراوح بين 3.6 و6.6 دولاراً أمريكياً. تراوحت تكلفة إجراء الاختبار التشخيصي السريع للجلوكوز 6 فوسفات ديهيدروجينيز، ما بين 0.9 إلى 13.5 دولارًا أمريكيًا، وهي أقل بشكل متسق عن تكاليف اختبار بقعة الفلورسنت المستخدم بشكل شائع (6.3 دولار أمريكي إلى 17.4 دولاراً أمريكياً).

**الاستنتاج:**

تؤدي نوبة الإصابة بملاريا *بلازموديوم فيفاكس * إلى ارتفاع التكاليف الأسرية. تعتبر تكاليف تشخيص وعلاج *بلازموديوم فيفاكس * مدخلات مهمة لتحليلات فعالية التكلفة المستقبلية لضمان تخصيص الموارد على النحو الأمثل للتخلص من الملاريا. ****

## 摘要

**目的:**

确定多个流行国家*间日疟原虫*感染带来的家庭成本和医疗成本。

**方法:**

我们收集了从 2014 年 4 月至 2017 年 12 月在阿富汗、埃塞俄比亚、秘鲁、巴西、菲律宾、哥伦比亚、泰国、印度尼西亚和越南进行的三项*间日疟原虫*治疗多中心临床试验的成本数据。我们从试验参与者在注册时进行的调查中推导出家庭成本,并在两周后再次进行调查以确定治疗和交通成本,以及患者及其家庭护理人员无法正常开展日常活动的天数。我们通过对研究点用于诊断和治疗*间日疟原虫*的资源进行了明细成本计算,确定了医疗护理提供者的一般性医疗成本。

**结果:**

各国的平均家庭总成本分别从阿富汗的 8.7 美元(美元;标准差:4.3)至哥伦比亚的 254.7 美元(标准差:148.4)不等。在所有国家中,生产力下降是家庭成本中最主要的部分,从而导致平均间接成本从 5.3 美元(标准差:3.0)至 220.8 美元(标准差:158.40)不等。医疗护理提供者进行一般性医疗的费用范围是 3.6–6.6 美元。进行葡萄糖-6-磷酸脱氢酶快速诊断测试的费用从 0.9 美元至 13.5 美元不等,比普遍使用的荧光点试验的费用(6.3 美元至 17.4 美元)要低。

**结论:**

*间日疟原虫*的发作会给家庭带来较高成本。诊断和治疗*间日疟原虫*的成本费用是未来进行成本效益分析的重要信息,以此来确保消灭疟疾的最佳资源分配。****

## Резюме

**Цель:**

Определение затрат поставщиков услуг в сфере здравоохранения и семей пациентов при всплесках малярии, вызванной *Plasmodium vivax*, в странах с различным уровнем заболеваемости малярией.

**Методы:**

Авторы собрали данные по затратам для трех многоцентровых клинических исследований по лечению инфекции *P. vivax* в Афганистане, Бразилии, Вьетнаме, Индонезии, Колумбии, Перу, Таиланде, на Филиппинах и в Эфиопии за период с апреля 2014 года по декабрь 2017 года. Затраты семьи определялись по данным анкетирования участников клинического исследования, проводившегося в момент включения в исследование и 2 недели спустя для определения затрат на лечение и проезд, а также для определения количества дней, в течение которых пациенты и ухаживавшие за ними члены семьи были не в состоянии вести обычный образ жизни. Затраты медицинских учреждений на обеспечение стандартного набора медицинских услуг оценивались посредством микрокалькуляции расходов на ресурсы, необходимые для диагностики и лечения инфекции *P. vivax* в центрах проведения исследований.

**Результаты:**

Средняя общая величина затрат семьи находилась в диапазоне от 8,7 долл. США (среднеквадратичное отклонение, СО 4,3) в Афганистане до 254,7 долл. США (СО 148,4) в Колумбии. Во всех странах потеря трудоспособности составляла наибольший компонент затрат семьи, при этом средние косвенные затраты составляли от 5,3 долл. США (СО 3,0) до 220,8 долл. США (СО 158,4). Затраты медицинских учреждений на обеспечение стандартного набора медицинских услуг составляли от 3,6 до 6,6 долл. США. Затраты на проведение экспресс-теста для диагностики заболевания по глюкозо-6-фосфат-дегидрогеназе составляли от 0,9 до 13,5 долл. США, что было во всех случаях ниже затрат на широко используемый тест флуоресцентного пятна (от 6,3 до 17,4 долл. США).

**Вывод:**

Вспышка заболеваемости малярией, вызванной *P. vivax*, дорого обходится семьям пациентов. Затраты на диагностику и лечение *P. vivax* должны обязательно учитываться в последующих анализах эффективности затрат с целью обеспечения оптимальных ресурсов для искоренения малярии.

## Introduction

Outside Sub-Saharan Africa, *Plasmodium vivax* is now the predominant cause of malaria, affecting 14.0 million patients in 2016.^1^ While cost–effectiveness analyses can inform the efficient provision of health-care interventions, information on the costs for providers, patients and their households should be available. The costs of *P. vivax* malaria infection are potentially different from those infection caused by *P. falciparum*, since *P. vivax* forms liver stages (hypnozoites), which lie dormant for weeks or months after the primary infection before reactivating to cause symptomatic infections (relapses). Hence, one infectious mosquito bite can result in multiple episodes, with the risk and frequency of relapses varying significantly between geographical regions.^2^

Accordingly, the clinical management of patients with *P. vivax* requires a radical cure with a drug regimen that kills both the blood and liver stages of the parasite. The only widely available compound with activity against the dormant liver stages is primaquine, which is usually prescribed as a 14-day regimen.^3^ The efficacy of primaquine is dependent upon the total weight-adjusted dose administered.^4^^,^^5^ Patents’ adherence to 14-day primaquine courses varies considerably^6^ and failure to complete a full course is common.^4^ Therefore, adherence and effectiveness can potentially be improved by administering the same total dose while reducing the duration of treatment. In a recent multicentre clinical trial, the safety and efficacy of a 7-day primaquine regimen was compared with a 14-day primaquine and placebo regimen (short-course primaquine trial).^7^ Clinical trials have also been conducted of tafenoquine compared to a 14-day primaquine and placebo regimen (tafenoquine trials).^8^^,^^9^ Tafenoquine is a single-dose drug for radical cure of malaria that has recently received regulatory approvals in Australia and the United States of America, with further submissions underway in malaria-endemic countries. Both drugs can cause haemolysis in individuals with glucose-6-phosphate-dehydrogenase (G6PD) deficiency, an inherited enzymopathy. For this reason, the World Health Organization (WHO) treatment guidelines recommend that, where possible, G6PD status should be ascertained in all patients before administration.^10^ Due to operational and financial constraints, screening for G6PD deficiency is rarely offered.^11^ This may change as new rapid diagnostic tests become more widely available to facilitate point-of-care testing. For G6PD-deficient individuals, an 8-week course of weekly primaquine doses is recommended^3^ with monitoring for anaemia.

Little research has been conducted on the costs of diagnosing and treating *P. vivax* malaria. In a recent review,^12^ only nine relevant studies were identified with varying percentages of cases due to *P. vivax*. Given the increasing options available for testing and radical cure treatment, cost surveys were included in the study design of the short-course primaquine trial^7^and adapted to the tafenoquine trials.^8^^,^^9^ We aimed to describe the costs of treating uncomplicated *P. vivax* malaria in a range of endemic settings using data from both households and health-care providers, collected alongside these trials. The results can inform economic evaluation and policy-making for *P. vivax* malaria testing and treatment strategies.

## Methods

### Household costs 

We collected household cost data at study sites in nine countries: Afghanistan, Brazil, Colombia, Ethiopia, Indonesia, Philippines, Peru, Thailand and Viet Nam (Box 1). Using questionnaires translated into local languages, patients, or informants on the patients’ behalf, were interviewed by study staff at enrolment (day 0) and again 2 weeks later. We analysed data on the direct and indirect costs to the patient’s household for individuals with *P. vivax* malaria. Here, we report only the costs from the first episode of malaria, to minimize bias arising due to changes in treatment-seeking behaviour that might result from being enrolled in the study.

Box 1 **Clinical trial details in the multicentre study of the economic costs of *P. vivax* malaria episodes**

The design of the Improving the Radical Cure of Vivax trial has been previously described.^7^ The trial was conducted from 20 July, 2014 to 31 December, 2017. Outpatients in Afghanistan, Ethiopia, Indonesia and Viet Nam were treated with locally recommended schizontocidal treatment (chloroquine or dihydroartemisinin–piperaquine) and randomized to receive either 14 days of primaquine (total dose 7 mg/kg), 7 days of primaquine (total dose 7 mg/kg) followed by 7 days of placebo or 14 days of placebo. Patients were screened for glucose-6-phosphate-dehydrogenase (G6PD) deficiency before enrolment, and individuals with G6PD deficiency were excluded from the randomized study and given standard schizontocidal treatment plus eight doses of weekly primaquine. All patients gave informed consent before enrolment. Follow-up was 12 months.

In the Global Assessment of Tafenoquine Haemolytic Risk trial^8^ and Dose and Efficacy Trial Evaluating of Chloroquine and Tafenoquine In Vivax Elimination trial,^9^ adult patients (≥ 17 years) with *P. vivax* malaria from Brazil, Cambodia, Colombia, Ethiopia, Peru, Philippines, Thailand and Viet Nam were treated with either 14-day primaquine (3.5 mg/kg) or single-dose tafenoquine (300 mg) and followed for 6 months. These trials were conducted from April 2014 to November 2016. No patients in Cambodia completed the cost survey. Patients were excluded if G6PD enzyme activity was less than 70%. In both these trials, patients were hospitalized for days 1–3 to evaluate drug safety; the costs related to this stay are not included here.

The surveys used to collect household cost data were similar for all trials, with some minor differences. The short-course primaquine trial asked about travel and payments for the current clinic while the tafenoquine trials asked about facilities where patients were diagnosed. For patient and caregiver time off due to illness, short-course primaquine surveys asked about usual activity whereas the tafenoquine trials asked about usual work. Patients specified the number of days off for themselves and caregivers in the short-course primaquine trial, whereas the tafenoquine trials had one category for more than 7 days. The tafenoquine trials did not ask what activity caregivers would normally be doing. The follow-up visit was on day 15 for the tafenoquine trials. For the short-course primaquine trial, this was day 13 for G6PD normal patients and day 14 for those with G6PD deficiency.

Direct costs included all out-of-pocket expenditure for the entire episode for the patient’s household: (i) treatment seeking: the cost of treatment-seeking before enrolment in the study (medications, tests and fees); (ii) transport for treatment seeking: the cost of transportation for treatment seeking before enrolment in the study; (iii) visit cost: payments for treatment at the study health-care facility (medications, tests and fees); and (iv) transport cost: the cost of transportation to the health-care facility (doubled to account for return journeys). We excluded costs related to the clinical study, such as monitoring visits to ensure primaquine adherence. 

Indirect costs consisted of productivity losses due to illness for patients and, where applicable, their caregivers. Patients reported the length of time they could not perform usual activities due to illness and the length of time caregivers stopped doing usual work to care for them. We summed the number of days and multiplied this by the gross domestic product (GDP) per capita per day in that country for 2016^13^ to calculate the total indirect costs of illness; we applied this adjustment regardless of whether the usual activities were paid or unpaid work, or days off school. We did not collect data on household income, wealth or education.

### Provider costs

We collected data on health-care provider costs only at four study sites in the short-course primaquine trial: Jalalabad in Afghanistan, Arba Minch in Ethiopia, North Sumatra in Indonesia and Dak-O in Viet Nam. Using data from clinic records and interviews with providers, we collected all costs of routine care and of potential additions to care for patients with *P. vivax* malaria, but not the costs of care related to the trial. Cost items included blood draws (finger prick or venous), malaria diagnosis (rapid diagnostic test and microscopy) and treatment (blood-stage and liver-stage), G6PD tests (rapid diagnostic test and fluorescent spot test) and the HemoCue test (HemoCue AB, Ängelholm, Sweden) for anaemia. G6PD testing was not routine at any of the sites, so we calculated the cost of rapid diagnostic test and fluorescent spot test as additional costs. The site in Ethiopia was a research facility, so we adjusted inputs to reflect a routine clinical care setting.

We made the following assumptions about provider costs. One blood draw per patient would be sufficient for all tests undertaken. Since the throughput of malaria patients was generally low at all facilities, the fluorescent spot test would be used as a point-of-care test (i.e. only one test run in a batch). The fluorescent spot test consumables would be stored in a freezer (or a refrigerator with a −20 °C freezer compartment) as this offered a more conservative estimate of long-term costs. The annual number of malaria patients per facility was used for equipment throughputs. We combined the country-specific costs of diagnosis and treatment for *P. vivax* malaria with the cost of patient visits using the WHO global cost database for an outpatient centre with beds.^14^

### Analysis

We report all costs in United States dollars (US$) for the year 2016. We collected costs using local currencies, then adjusted them for inflation if from a different year,^15^^,^^16^ before conversion to US$.^17^ We also converted key unit costs to international dollars.^18^ For the tafenoquine studies, we used the midpoint of recruitment as the cost year for patient-level data. We analysed patient-level data using Stata, version 14.2 (StataCorp, College Station, USA). We excluded patients who did not complete a cost survey at both the initial and follow-up visits from the analysis. To present the costs of an uncomplicated malaria episode, we excluded patients reporting inpatient hospitalizations that are indicative of severe illness. For patients meeting the inclusion criteria, missing data for costs were minimal (< 0.1%) and assumed to be zero except for three variables in the tafenoquine studies. The first two were the number of days lost by patients and caregivers at one of the trial sites in Brazil.^8^ Since these were not collected, we imputed the mean time losses in Brazil from the other trial^9^ for missing data. The second exception was the visit costs where no entries were made: we assumed these costs were zero since treatment fees were paid by the study. For patients in the tafenoquine studies who reported more than 7 days of lost productivity, we assumed they had lost 8 days.

All data are presented by country, pooling data from Ethiopia and Viet Nam, which were included in all the trials. We report the mean, standard deviation (SD), median and interquartile ranges for household-level costs and days lost per episode of malaria. For provider costs, point estimates are presented. The incremental costs of switching from fluorescent spot test to G6PD rapid diagnostic test are reported.

### Ethical approval

Ethical approval for the original Improving the Radical Cure of Vivax Malaria protocol and included amendments (Version 7) was obtained from the following review boards: Oxford Tropical Research Ethics Committee OxTREC (Ref number 101413) and the Human Research Ethics Committee of the Northern Territory Department of Health, Australia HREC (Ref Number 131991). In addition local approvals were obtained from the Institutional Review Board, Ministry of Public Health, Afghanistan, the Health Research Ethics Committee, Faculty of Medicine University of Indonesia, Cipto Mangunkusumo Hospital, Jakarta, Indonesia, the Ministry of Health Evaluation Committee on Ethics in Biomedical Research Vietnam, the Institutional Scientific & Ethical Review Committee of the Ethiopian Public Health Institute, the National Research Ethics Review Committee, Ethiopia and the Institutional Review Board of the Columbia University Medical Centre, US. The Global Assessment of Tafenoquine Haemolytic Risk trial (NCT 02216123) and Dose and Efficacy Trial Evaluating of Chloroquine and Tafenoquine In Vivax Elimination (NCT 01376167) studies are registered.

## Results

### Patient characteristics

**Fig. 1:**
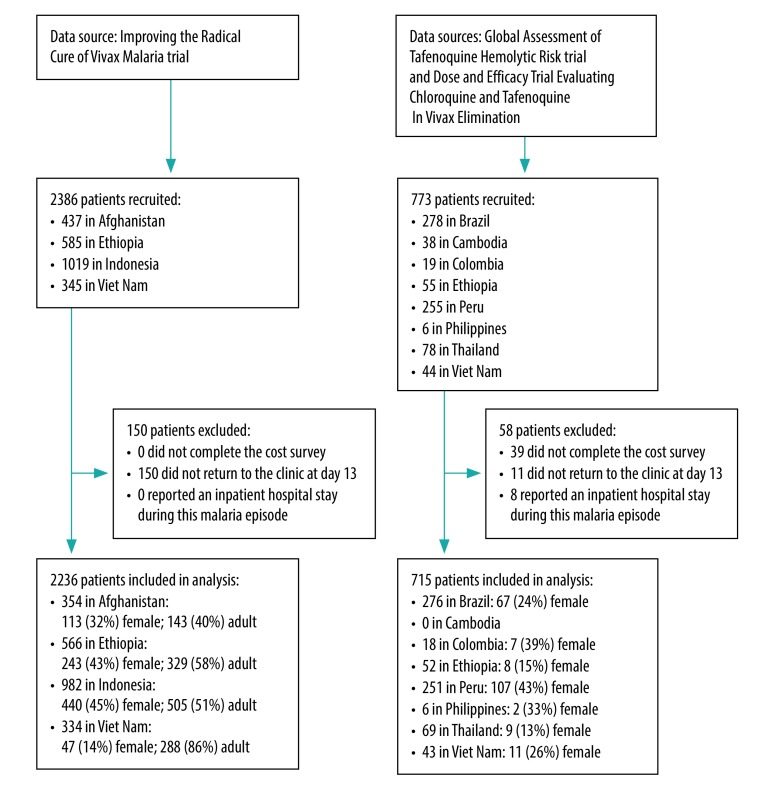
Flow diagram of participants selected from three multicentre clinical trials of *P. vivax* malaria treatment

Notes: Adult was defined as age ≥ 15 years.

Of 3159 patients enrolled in the studies, 2951 (93%) completed cost surveys at both the initial and follow-up visit: 2236 patients from the short-course primaquine trial and 715 from the tafenoquine trials (Fig. 1). Most patients were male (1897; 64%) and ≥ 15 years old (1980; 67%). The percentage of enrolled females in the tafenoquine trials (211 out of 715; 30%) was lower than in the short-course primaquine trial (843/2236, 38%). In the short-course primaquine trial, 43% (971/2236) were children aged < 15 years, with the proportion of children varying by country from 14% (46/334) in Viet Nam to 60% (211/354) in Afghanistan (Fig. 1). Since children younger than 17 years were excluded from the tafenoquine trials, only 33% (971/2951) of patients overall were children. Patients ranged in age from 10 months to 94 years.

Data source: Taylor WRJ et al,^7^ Llanos-Cuentas A et al.,^8^ and Lacerda MVG et al.^9^

### Household costs

#### Productivity losses

Patients reported a mean loss of 3.5 days (SD: 3.1) from their usual activities over the entire episode of malaria (Table 1). More details of the data are available from the data repository.^19^ Of the 2883 patients who reported their usual activities, 1007 (35%) would have been at school, 436 (15%) were subsistence farmers, 391 (14%) missed paid employment, 378 (13%) would have been doing housework and 671 (23%) reported another activity. Most patients (78%, 2243/2883) reported needing someone to cut back on usual activities to care for them, with a mean loss of 1.8 days (SD: 2.8). In Viet Nam, 59% (221/377) of patients reported needing a caregiver, compared with 98% in both Brazil (204/208) and Peru (247/251). The highest mean number of lost days was in Colombia and the Philippines for both patients and caregivers (Table 1). Half (823/1633) of caregivers from the short-course primaquine trial reported that they would normally be doing housework, 216 (13%) were unable to farm, 101 (6%) missed school, 179 (11%) missed paid employment and 314 (19%) reported that they would be doing another activity (the tafenoquine trials did not ask about usual activities for caregivers). Further information on activities by country is available from the data repository.^20^ When multiplied by the GDP per capita for each country, these productivity losses resulted in mean indirect costs that ranged from US$ 5.3 (SD: 3.0) per episode in Afghanistan to US$220.8 (SD: 158.4) in Colombia (available from the data repository).^21^

**Table 1:** Productivity losses to patients and their caregivers due to an episode of *P. vivax* malaria illness in malaria-endemic settings in nine countries, 2014–2017

Country	No. of clinical episodes	No. of days patient unable to do usual activities			No. of days caregiver was required	
		Mean (SD)	Median (IQR)		Mean (SD)	Median (IQR)
Afghanistan	354	2.2 (1.3)	2.0 (1.5–3.0)		1.2 (1.1)	1.0 (0.0–2.0)
Brazil	276	1.3 (1.9)	1.0 (0.0–1.3)		0.2 (0.8)	0.0 (0.0–0.2)
Colombia	18	7.0 (5.4)	7.0 (3.0–11.0)		7.0 (5.2)	6.0 (3.0–11.0)
Ethiopia	618	3.3 (2.5)	2.5 (2.0–4.0)		2.2 (2.6)	1.5 (0.0–3.0)
Indonesia	982	3.8 (3.7)	3.0 (0.5–5.0)		2.7 (3.6)	1.0 (0.5–4.0)
Peru	251	6.3 (2.5)	6.0 (5.0–8.0)		0.5 (0.2)	0.5 (0.5–0.5)
Philippines	6	9.8 (5.2)	10.0 (4.0–15.0)		4.3 (3.9)	3.0 (1.0–8.0)
Thailand	69	4.1 (2.7)	5.0 (2.0–6.0)		1.9 (2.6)	1.0 (0.0–4.0)
Viet Nam	377	3.2 (2.5)	2.5 (1.5–4.0)		1.2 (1.8)	0.5 (0.0–1.5)
**Overall**	**2 951**	**3.5 (3.1)**	**3.0 (1.0–5.0)**		**1.8 (2.8)**	**1.0 (0.0–2.0)**

IQR: interquartile range; SD: standard deviation.

Notes: We collected data alongside three multicentre clinical trials of *P. vivax* malaria treatment from April 2014 to December 2017.^7^^–^^9^Patients reported the length of time they could not perform usual activities due to illness and the length of time caregivers stopped doing usual activities to care for them. We pooled data from different trials in Ethiopia and Viet Nam. More details are available from the data repository.^19^

#### Treatment-seeking costs

Overall, 1671 (57%) of the 2951 patients were seeking treatment for the first time. This proportion ranged widely by country from 0% (0/251) in Peru to 91% (322/354) in Afghanistan (available from the data repository).^22^ For this episode of malaria, 659 patients (22%) had sought treatment once before, 598 (20%) had sought treatment twice before and 23 (1%) had sought treatment three times before the enrolment visit. While many patients reported spending a mean of US$ 2 or less, patients in Colombia and Thailand spent a mean of US$ 19.2 (SD: 27.1) and US$ 22.0 (SD: 39.2), respectively (available from the data repository).^21^

While transportation to the study clinic cost over US$ 10 for a return journey in Brazil, Colombia and Thailand, these costs were low in most countries. Overall, 93% (2745/2951) of patients reported a journey of under 1 hour. The majority of those with long travel times were in Afghanistan, where 28% (99/354) reported journeys of over 1 hour to get to the clinic, with correspondingly higher mean transportation costs of US$ 3.1 (SD: 1.5).

Considering all transport and treatment costs, the mean direct household expenditure was US$ 6.6 (SD: 12.2), varying from US$ 1.6 (SD: 2.2) in the Philippines to US$ 35.3 (SD: 46.6) in Thailand (available from the data repository).^21^

#### Total household costs

The mean total costs to households due to illness (indirect costs from lost productivity plus direct costs of transportation and treatment) varied by country, ranging from US$ 8.7 (SD: 4.3) in Afghanistan to US$ 254.7 (SD: 148.4) in Colombia (available from the data repository).^21^ Costs were higher for women in Afghanistan, Ethiopia, Indonesia and Viet Nam and for men in Brazil and Peru. Viet Nam was the only country where no age-related differences were apparent, a likely reflection of nearly all patients being older than 15 years (available from the data repository).^23^

### Provider costs

The total cost of routine care by health-care providers per patient visit in the four settings ranged from US$ 3.6 in Afghanistan to US$ 6.6 in Indonesia (Table 2). The cost per test and associated laboratory processing time are available from the data repository.^24^ In these settings with relatively low patient throughputs, implementing fluorescent spot testing resulted in a cost range of US$ 6.3–17.4 compared with US$ 0.9–13.5 for G6PD rapid diagnostic tests. Some settings reported that fluorescent spot tests and G6PD rapid diagnostic tests had to be repeated for some patients, thereby increasing the costs. G6PD rapid diagnostic testing was consistently cheaper per patient than fluorescent spot testing, even in Indonesia where the unit cost of a G6PD rapid diagnostic test was US$ 13.0. Potential cost savings per test using a G6PD rapid diagnostic test instead of the fluorescent spot test ranged from US$ 3.5 in Indonesia to US$ 16.1 in Viet Nam. The cost of monitoring for anaemia with the HemoCue test ranged from US$ 1.2 in Viet Nam to US$ 3.0 in Ethiopia.

**Table 2:** Costs to health-care providers of routine care for a patient with *P. vivax* malaria illness in four malaria-endemic settings

Care item	Cost per patient, US$			
	Afghanistan	Ethiopia	Indonesia	Viet Nam
Patient visit	1.68	0.98	0.08	1.74
Malaria diagnosis	1.14	3.51	2.86	3.47
Blood-stage drugs	0.28	0.36	2.65	0.27
Liver-stage drugs	0.19	NA^a^	0.43	0.43
Other medications	0.33	0.43	0.59	0.09
**Total cost**	**3.62**	**5.28**	**6.61**	**6.01**

NA: not applicable; US$: United States dollars (2016).

^a^ Liver-stage drugs are not included as routine care in Ethiopia.

Notes: We collected data on health care provider costs on the typical costs per *P. vivax* malaria patient alongside a multicentre clinical trial in the study countries.^7^ Basic visit costs were taken from the World Health Organization CHOICE unit cost estimates for service delivery at an outpatient centre with beds.^14^Diagnosis costs included microscopy in Afghanistan, Ethiopia and Viet Nam, plus rapid diagnostic test for malaria in Indonesia and Viet Nam. Costs of blood-stage drugs included dihydroartemisinin–piperaquine in Indonesia and chloroquine in Viet Nam. In Afghanistan, 90% received chloroquine tablets and 10% received chloroquine syrup. In Ethiopia, 78% of patients received chloroquine tablets, 20% received chloroquine syrup and 2% of patients received artemether–lumefantrine. Liver-stage drugs included low-dose primaquine for all patients in Indonesia and Viet Nam, while 50% of patients received low-dose primaquine in Afghanistan. Costs of other medications included drugs frequently prescribed for malaria (but not antimalarials) were multiplied by the percentage of patients receiving those medications. All drug costs are for adult prescriptions. More details of the test costs are available from the data repository.^24^

### Total costs

**Fig. 2:**
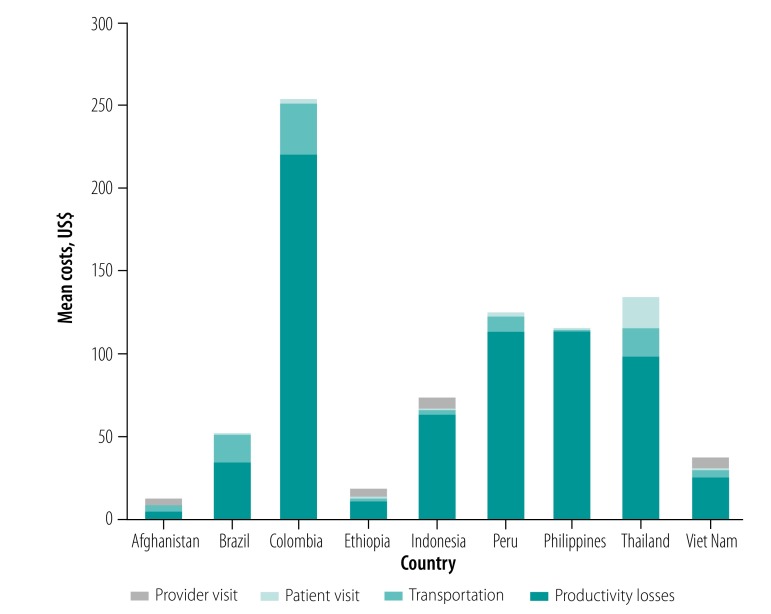
Total mean costs per episode of *P. vivax* malaria in malaria-endemic settings in nine countries

Fig. 2 summarizes the total costs per *P. vivax* malaria episode in the nine countries, including all provider (direct) costs and the household direct and indirect costs. As shown, the indirect household costs due to productivity losses were consistently the largest contributor to overall costs.

US$: United States dollars (2016).

Notes: We collected household and provider costs using local currency and adjusted to 2016 US$.^15^^–^^17^ Cost components include household visit costs (including treatment seeking) and transportation (for the visit and treatment seeking), indirect costs to households (productivity losses) and direct costs of health-care providers (visit including diagnosis and treatment of *P. vivax* malaria). Household cost data were collected alongside three multicentre clinical trials of *P. vivax* malaria treatment.^7^^–^^9^ Numbers of patients surveyed: Afghanistan: 354; Brazil: 276; Colombia: 18; Ethiopia: 618; Indonesia: 982; Peru: 251; Philippines: 6; Thailand: 69; Viet Nam: 377. Data for estimating health-care provider costs were available from one clinical trial.^7^

## Discussion

Our study presents a large collection of primary cost data for the treatment of *P. vivax* malaria, incorporating information from both households and health-care providers from a range of endemic countries. Unlike previous studies,^12^ the costs reported here are due solely to individuals infected with *P. vivax*. The mean total household cost per episode varied considerably (from around US$ 9 to US$ 255), reflecting differences in both the duration of time off normal activities and GDP per capita in the country. When productivity losses are excluded, these costs fall to around US$ 2 to US$ 35. While time is a scarce resource with economic value, productivity losses are rarely included in economic evaluations as costs to society as whole (i.e. including costs to patients and their households) are not often presented.^25^^,^^26^ While the costs from the health-care provider perspective is appropriate for priority-setting within the health sector, indirect productivity losses can be the larger cost component for households.^27^ Even when days lost to illness and caregiving are captured by surveys, the cost of these days can be difficult to value.^28^ The impact of ill health on members of the household (spillover effects) is a rapidly evolving area in health economics and a consensus has not been reached on whether to include spillover effects in economic evaluations, even when taking the societal perspective.^29^^,^^30^ A recent review on measuring and valuing time losses due to informal caring demonstrates that much of the thinking in this area has been focused on chronic conditions in developed nations.^31^ Changing the scope of economic evaluations could exacerbate inequities in health and access to health-care technologies.^28^ Our results rely on revealed preference methods that focus on the monetary value of time losses to the caregivers without consideration of (dis)utility due to the patients’ illness; accordingly, there is no risk of double counting.^31^

The most common approach for valuing the time of those not in paid employment is to collect information on daily wages for those in paid employment and use the average cost as a shadow wage. Most patients in this study were not in paid employment, so this approach would provide limited data. Reassuringly, a study in Timika, Indonesia found a shadow wage of US$ 11 (2014), demonstrating that our use of one GDP per capita per day of US$ 10 (2016) may be conservative. Similar methods of valuation are being implemented in other global health studies,^32^^,^^33^ and it is important to note that this figure is equivalent to 1/365th of a disability-adjusted life-year when using a willingness-to-pay threshold of one GDP per capita.

Malaria exerts a greater burden on poorly resourced communities and families who are likely to have lower shadow wages; hence, the use of GDP per capita per day for lost wages may have overestimated the indirect costs of *P. vivax* malaria. Days lost to illness can be catastrophic for a family reliant on subsistence farming and may contribute to a cycle of poverty. We assumed no one else within a household made up the lost income, although this could be a mitigating strategy.^34^ The valuation of productivity losses may be overestimated for children; however, this would be offset by decreased performance at school,^35^^,^^36^ which were excluded. A study in Ethiopia also found significant differences in indirect and total costs of care by malaria species.^37^ Since *P. vivax* malaria is a disease epitomized by recurrent episodes and long-term morbidity with limited direct mortality, quantifying and valuating these productivity losses are essential for a comprehensive analysis of the economic burden of the disease.^38^^,^^39^ Indeed, previous research indicated that countries with a high percentage of the population at risk of *P. falciparum* infection were likely to experience reductions in growth as great as 2% GDP per capita.^40^

Testing for G6PD deficiency, as recommended by WHO,^3^ resulted in additional costs ranging from US$ 1 for the rapid diagnostic test in Ethiopia and Viet Nam to US$ 17 for the fluorescent spot test in Indonesia and Viet Nam. At US$ 6–17 per test, the fluorescent spot test is an expensive option. Due to the limited shelf life of the reagents, the cost per patient will increase as the numbers of cases at a facility decrease. While this would be less expensive in the short-term, the overall cost per test would increase as the reagents last twice as long in the freezer unless the refrigerator has a −20 °C compartment as the facilities in Ethiopia and Viet Nam did. While the G6PD rapid diagnostic test was consistently less expensive than the fluorescent spot test, the cost savings per test given were dependent on the in-country costs of consumables, ranging from US$ 4 per person in Indonesia to US$ 16 in Viet Nam. In Indonesia, where primaquine is prescribed without G6PD testing, the incremental cost per person treated is likely to be a major obstacle to convincing policy-makers to implement routine testing. Furthermore, if primaquine treatment is to be routinely administered, the use of a HemoCue test (or equivalent) might be viewed as necessary to monitor patients for anaemia. While it is a relatively inexpensive test (US$ 3 or less), it may be required more than once per *P. vivax* episode.

This study has several limitations. Household visit costs may have been lower in a research context than in routine care. Cost data were missing for 7% of the patients in the short-course primaquine trial and 8% of patients in the tafenoquine trials, potentially creating a bias. For example, fewer patients in Afghanistan returned at day 13, which could reflect higher travel costs for those individuals. Furthermore, the tafenoquine trials required hospitalization, which may have created a bias in those who enrolled. While we have data from a large and diverse set of sites in nine countries, these sites may not fully reflect the considerable heterogeneity within countries. Productivity losses were a key driver of the total costs, but there is no consensus on how best to valuate these, particularly for children in whom the incidence of *P. vivax* malaria is highest in many settings.

In conclusion, the economic burden of *P. vivax* malaria is substantial at the household level, with the largest component arising from lost productivity. Ensuring safe radical cure through G6PD rapid diagnostic testing would reduce this burden, but uptake of tests may be impeded if associated with high test costs. We propose that this large collection of *P. vivax* malaria costs is an important resource with which to embark upon more robust cost–effectiveness analyses in the future.
